# Pre-Clinical Study: Immunohistochemical evaluation of matrix metalloproteinase-13 on rabbit ( *Oryctolagus cuniculus)* socket healing after application of platelet-rich fibrin with and without hydroxyapatite

**DOI:** 10.12688/f1000research.74094.1

**Published:** 2022-01-11

**Authors:** Meta Maulida Damayanti, Meike Rachmawati

**Affiliations:** 1 Pathology Anatomy, Universitas Islam Bandung, Unisba, Bandung, West Java, 40116, Indonesia

**Keywords:** Socket Healing, Matrix Metalloproteinases-13, Platelet-Rich Fibrin, Hydroxyapatite

## Abstract

**Background:** Tissue engineering technology has been used globally and proven to accelerate wound healing. This study aimed to analyse the effect of adding hydroxyapatite (HA) as a scaffold to platelet-rich fibrin (PRF) as a growth factor in accelerating the wound healing process as seen from the expression of matrix metalloproteinase-13 (MMP-13).

**Methods:** This research is an animal experiment conducted on 18 rabbits ( *Oryctolagus cuniculus)*. Rabbits were randomly divided into the following three groups of treatment: (G1) the application of PRF group, (G2) the application of PRF+HA group and (C) the control group without any application. Furthermore, each treatment group was split randomly into three groups of observation time. Periodontal tissue biopsy was performed to analyse the histopathological features that were examined on the basis of the level of MMP-13 immunoexpression.

**Results:** MMP-13 immunoexpression in the PRF+HA group showed better histoscore results, indicating a substantial reduction in MMP-13 values compared with other groups. The healing process was shown to increase with increasing observation time (p<0.05), and the PRF+HA group outperformed the PRF and control groups. On day 3, MMP-13 exhibited a dark brown colour of Immunohistochemistry (IHC), which indicated an increase in the expression value of MMP-13 in the early stages of healing, namely, inflammation. On day 14, light brown IHC was seen, especially in group 2, as a reference that the remodeling process had begun.

**Conclusions:** This study indicates that the application of HA can accelerate the socket healing process by decreasing the level of immunoexpression of MMP-13. HA is an alloplastic material that has inherent bioactive properties that support osteoconduction, which functions as a scaffold in the form of a fibrin matrix that can bind MMPs so that it can accelerate the wound healing process.

## Introduction

Tooth extraction is one of the most common treatment procedures in dentistry. This procedure may cause resorption of the alveolar bone, gingival recession around the extraction area and periodontal abnormality and may also result in an aesthetic problem because of post-extraction anatomical and physiological changes. ^1^^,^^2^ The wound healing process is influenced by various molecules, inflammation mediators, integrins, growth factors and matrix metalloproteinases (MMPs). MMPs has a function in every phase of wound healing by making modifications to the wound matrix, enabling cell migration essential in the remodeling process. Furthermore, MMPs are responsible for collagen degradation, and tissue engineering is one of the treatment options for regenerating periodontal tissues and alveolar bone. Progenitor cells, scaffolds, and growth factors are three essential components of tissue engineering. ^3^^,^^4^

MMP-13 has an important role in the early diagnosis of arthritis by analysing its pathological activity on the basis of histopathological features. ^5^ Fibroblast cells are abundant in the stroma, and these cells play a role in making the extracellular matrix (ECM) function properly. During the wound healing, inflammatory, proliferative and remodeling stages, fibroblasts adapt to their environment and respond to and emit signals locally. At the time of injury, fibroblasts can replace the injured tissue. During pathological conditions, the ECM is produced in excess, and collagen is deposited irregularly, often leading to irreversible organ dysfunction or a disfiguring appearance. ^6^

Bone grafts are used to replace and regenerate the lost bone. Scaffolding materials studied have been widely employed to stimulate bone growth. A promising scaffold must have biomaterials similar to the original bone structure. ^7^ Platelet concentrates, such as platelet-rich fibrin (PRF), are utilised in different clinical fields, particularly in a medical procedure involving the mouth and jaws, the concentrates contain a high concentration of growth factors, which are essential in wound healing, particularly bone regeneration. Thus, they are considered as another treatment adjuvant. ^8^^–^^12^ Research that utilised platelets as growth factor therapy has affirmed that the use of PRF is better in wound healing compared with platelet-rich plasma (PRP), which is seen from various diagnostic tools. ^11^ In dentistry, PRF has proven to accelerate wound healing. ^13^

Hydroxyapatite (HA) is a fundamental part of hard tissues, namely, bones and teeth. HA (Ca _10_(PO _4_) _6_(OH) _2_) is a material to assist with bone regeneration, it has great biocompatibility, does not cause unnecessary inflammation phenomena. It is non-poisonous, has great osteoconduction, it also has a high affinity for binding with other materials, and is a manufactured material generally utilised in medication and dentistry. ^11^^,^^14^ In addition, HA can be employed as a scaffold in bone tissue regeneration. Growth factors and scaffold products induce the release of inflammatory molecules and mediators, such as tumour necrosis factor-alpha (TNF-α), interleukin (IL)-1, IL-6 and MMP-13, to render a better catabolic effect on chondrocyte metabolism and to speed up the inflammatory process. ^15^ Scaffold functions as an early ECM essential for cell proliferation, migration and differentiation, while growth factor has a vital role in the wound healing process, an acute tissue response to trauma and several cell physiological processes. This study aimed to analyse the effect of adding HA as a scaffold to PRF as a growth factor in accelerating the wound healing process as observed from the expression of MMP-13.

## Methods

### Ethical clearance

All experimental procedures involving animals were conducted in accordance with ARRIVE guidelines 2.0 on the care and use of laboratory animals to ameliorate any suffering for the animals. ^16^ The treatment of the experimental animals was according to the regulations regarding the convention on international trade in endangered species of wild fauna and flora. A research proposal containing research procedures was submitted to the ethics committee and passed the ethical committee issued by the University of Padjadjaran Bandung, Indonesia (Number: 132/UN6.C1.3.2/KEPK/PN/2017).

### Study design

This analytic, quantitative research used an animal experimental laboratory design, and a post-test-only control group study design was conducted. The sample size was determined using the Mead equation formula calculation. Subjects were 18 rabbits ( *Oryctolagus cuniculus*) chosen according to the following: healthy, good inferior anterior teeth and 300–400 g of weight. The animals were not selected on the basis of sex and age because they did not affect the treatment. The study employed three treatment groups and three observation times. The treatment groups were as follows: the control group (C), the PRF group (G1) and the PRF+HA group (G2), and the observation times were the following Day 3, Day 7 and Day 14 based on the inflammation, proliferation and remodeling phases of the healing process. Each animal obtained different treatments with distinct observation times. The experimental animals were randomly divided into three treatment groups and then split into three groups on the basis of the time of observation. Each group was marked on the back area using markers (C, G1 and G2) and placed in a different cage according to the time of observation. Cage A for Day 3, B for Day 7 and C for Day 14.

After subject selection, a rabbit adaptation was performed for 7 days, and all the animals fasted for 12 hours before tooth extraction. Thereafter, tooth extraction was performed, PRF was applied, and then HA was added. A commercial brand of HA, which is commonly used in dentistry treatment, was chosen for this study. During the experimental period, all the experimental animals were given standard feeds and cages met the ethical standards. All the research conditions and procedures were recorded daily by the team in their logbooks. Details such as group allocations, preparations, treatment, and data collection and analysis were duly noted.

### Platelet preparation

Three millilitres of homologous blood was acquired in sterile cylinders from the ear cartilage of each rabbit. From that point onward, the blood was quickly centrifuged at 3200 rpm (1600 g) for 10 minutes. The blood was isolated into three layers, and the top layer was separated from the other layers by transferring to another centrifugation tube and then was centrifuged again at 3200 rpm (1600 g) for 15 minutes. ^10^^,^^17^

### Tooth extraction process in animal models

Extraction was performed under anaesthesia (ketamine and xylazine) to relieve pain. During the extraction process, the experimental animals were conditioned as comfortably as possible and were put to sleep on a support board. The enclosed action area was not visible to other experimental animals. Further, the tooth extraction was executed with labial and lingual luxation movements using pedodontic forceps for the inferior anterior region with the principle of minimal injury. After the extraction, the socket was irrigated using a 0.09% saline solution and was drained using a sterile tampon. The socket was filled with PRF (G1) and PRF+HA (G2), and nothing was given to the control group. After the application was made, the wound was closed with simple stitches using a 3.0 silk stitches thread and a curved needle with a simple interrupted suture method. The periodontal tissue biopsy specimens were taken on the basis of the observation time (Days 3, 7 and 14). Termination was conducted before taking the specimens using ketamine at a dose of 200 mg/kg rabbit body weight. The limitation of this study is that, after tooth extraction, intervention was executed on the basis of the treatment group, and then simple stitches was performed. However, stitches can loosen up if the experimental animal is too active in moving its teeth and tongue, even making the stitches to come off.

### Immunohistochemical of MMP-13 processing and evaluation

Immunohistochemical tests were performed to analyse the immunoexpression of MMP-13. The current research used an MMP-13 primary antibody kit, a labelled streptavidin-biotin (LSAB) secondary antibody kit, polyclonal antibody (MyBioSource, Cat# MBS837431, RRID: AB_2895530), and positive control of breast cancer. The immunoexpression of MMP-13 appeared to be positive when the connective tissue/stromal fibroblast around was brown in colour by looking at the distribution and intensity. Histoscore/final score is the multiplication of distribution with intensity, and the values for the distribution are as follows: 4 = cells positive > 75%, 3 = cells positive 51%–75%, 2 = cells positive 25%–50% and 1 = cells positive < 25%. The value for intensity is 0 = no colour, 1 = current colour, weak (light brown), 2 = present colour, moderate (brown) and 3 = present colour, strong (dark brown). ^10^^,^^18^^,^^19^ This research was conducted in Laboratory Pathology Anatomy Hasan Sadikin General Hospital Bandung, Indonesia, and the immunoexpression scores of MMP-13 were evaluated by a qualified pathologist.

### Statistical analysis

Data analysis was conducted using the Statistical Package for Social Science (SPSS) software (IBM SPSS Statistics, RRID:SCR_019096). Normal distribution data analysis was performed using the Shapiro–Wilk test if normally distributed, and parametric tests were performed. The evaluation of MMP-13 among the groups and observation times was performed statistically with the Kruskal–Wallis test. All the data were uploaded to the repository. ^20^^–^^22^

## Results

The addition of HA to PRF displayed faster wound healing in the socket. The total histoscore in group 2 was lower than that in group 1 and the control group. Table 1 shows differences in MMP-13 immunoexpression between groups. Histoscore Days 7 and 14 had a significant difference (p < 0.05). On the third day, the MMP-13 values were the same in all the groups. On Day 7, the MMP-13 value began to decrease, where group 2 showed a significant decrease. On Day 14, it was observed that MMP-13 had a lower histoscore, especially in group 2.

**Table 1.** Histoscore difference of MMP-13 between groups (n=18).

MMP-13	Treatment Group			p-Value
	C *	G1 *	G2 *	
**Distribution**				
Day 3	4.00 (4.00-4.00)	4.00 (4.00-4.00)	3.75 (3.00-4.00)	0.368
Day 7	3.50 (3.00-4.00)	3.50 (3.00-4.00)	3.50 (3.00-4.00)	0.400
Day 14	3.00 (3.00-3.00)	4.00 (3.00-4.00)	3.00 (3.00-3.00)	0.111
Total	3.50 (3.00-4.00)	4.00 (3.00-4.00)	3.00 (2.00-4.00)	0.648
**Intensity**				
Day 3	3.00 (3.00-3.00)	3.00 (3.00-3.00)	3.00 (3.00-3.00)	1.000
Day 7	3.00 (3.00-3.00)	3.00 (3.00-3.00)	2.50 (2.00-3.00)	0.004
Day 14	3.00 (3.00-3.00)	2.00 (2.00-3.00)	2.00 (2.00-2.00)	0.026
Total	3.00 (3.00-3.00)	3.00 (2.00-3.00)	2.50 (2.00-3.00)	0.001
**Histoscore**				
Day 3	12.00 (12.00-12.00)	12.00 (12.00-12.00)	11.25 (9.00-12.00)	0.368
Day 7	11.25 (9.00-12.00)	9.75 (9.00-12.00)	7.00 (6.00-8.00)	0.013
Day 14	9.00 (9.00-9.00)	8.50 (8.00-9.00)	6.00 (6.00-6.00)	0.009
Total	11.25 (9.00-12.00)	10.00 (8.00-12.00)	8.00 (6.00-12.00)	0.011

* Median value (range).

**Figure 1.**
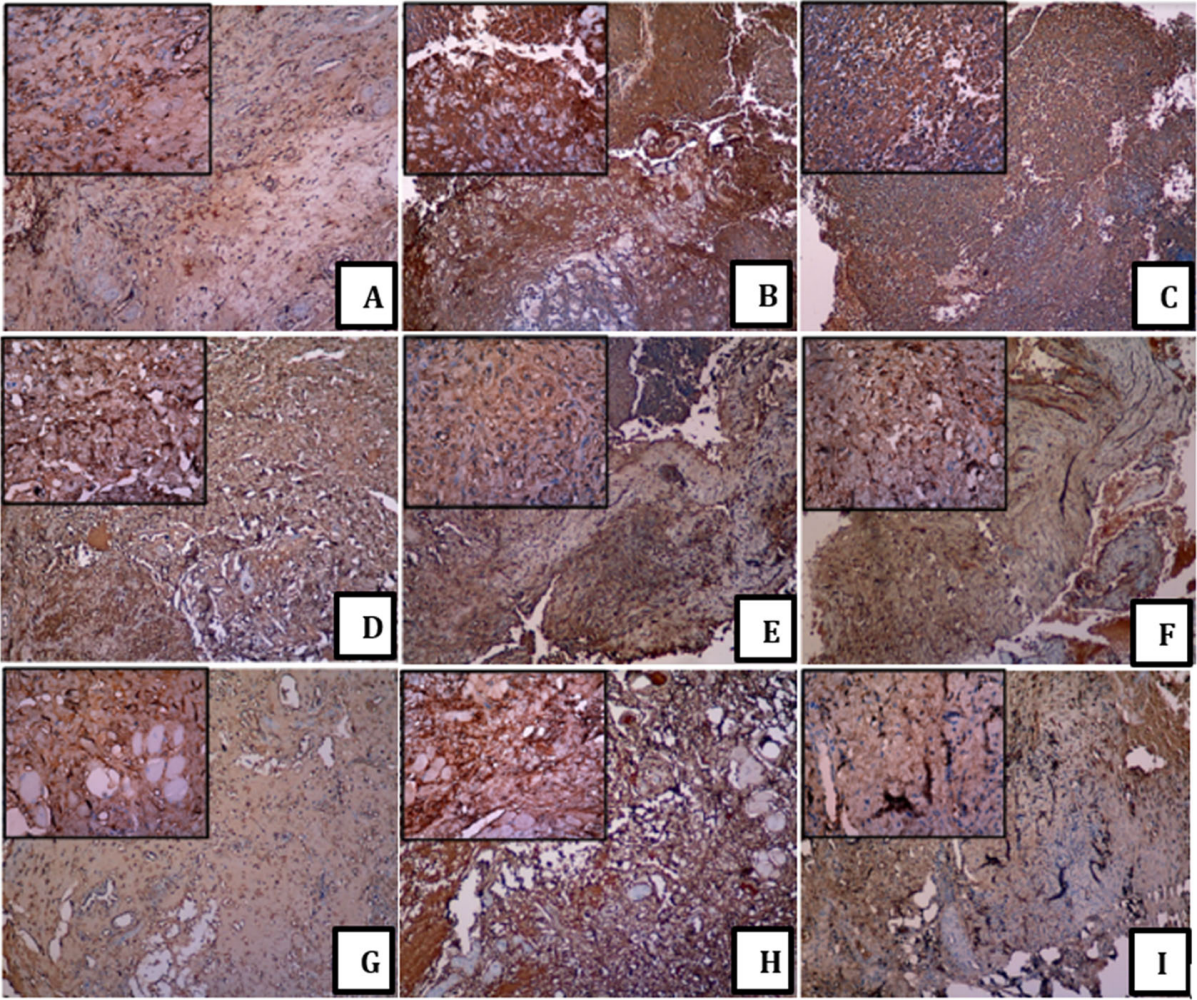
Immunohistochemistry of MMP-13 among a group. (A) control group day 3; (B) control group day 7; (C) control group day 14; (D) group 1 day 3; (E) group 1 day 7; (F) group 1 day 14; (G) group 2 day 3; (H) group 2 day 7; (I) group 2 day 14.

The brown staining addresses the showed antigen, while the blue colour was the counterstain of the cores. Socket healing featured MMP-13 protein in the cytoplasm of the stromal fibroblast cells. In addition, the undeniable levels of MMP-13 protein were solely in the cytoplasm of the fibroblast cells in all the groups for day 3 [ Figure 1 (A), (D) and (G)]. The light brown colour on fibroblast represented the low IHC staining of the MMP-13 expression (Days 7 and 14 and groups 1 and 2) [ Figure 1 (F), (H) and (I)]. The brown colour on the fibroblast represented the moderate IHC staining of MMP-13 expression (B), (C) and (E). The large and small image inserts exhibited the portion of each treatment group and the observation time at magnifications 100× and 400×. Furthermore, administration platelets increased the MMP-13 expression in line with HA levels. The histopathological picture showed the expression of MMP-13 with a dark brown appearance in the stromal fibroblast area, which was evenly distributed in all areas with strong intensity (A, D and G). In contrast to the IHC picture, which looked light brown, especially in pictures (F) and (I), it displayed a reduced distribution and intensity (see Figure 1). ^22^

Table 2 showed that administration of PRF and PRF+HA could reduce MMP-13 levels during the observation time. There was a significant difference in examination between observation times. Of all group, group 2 saw a significant difference based on the healing time.

**Table 2.** Differentiation of each treatment groups by observation times on Day 3, Day 7, and Day 14.

	P Value *
Treatment group	MMP-13
C	0.017
G1	0.016
G2	0.010

* Based on Kruskal Wallis test.

## Discussion

The present study validated that MMP-13 could determine healing activities. Hydroxyapatite is the primary mineral aspect of vertebrate bones and teeth, and it's far a good cloth for growing as a simple, efficient, and environmentally pleasant approach of forming biofunctional scaffolds and implant coatings with sizeable biocompatibility, bioactivity, mechanical strength, and the cap potential to feature as a drug delivery system. ^23^

This preclinical study confirmed the hypothesis that the application of HA in post-extraction socket healing can accelerate the healing process and decrease the level of MMP-13 immunoexpression based on the stages of wound healing, namely the stages of inflammation, proliferation, and progression. On Day 3, MMP-13 was highly expressed, proving that up to 4–5 days after an injury, there will be an inflammation phase. On the next day, the expression began to decrease, especially in group 2, meaning that HA could accelerate the work of growth factors in the wound healing process. On Day 14, there was a decrease in expression, indicating that the inflammatory process was finished and that tissue regeneration or the stages of proliferation and progression began. In this study, there was a significant difference based on the observation time from Day 3 to Day 14.

This result related to the theory that growth factors are an important part of the bone restoration response delivery system and delivering exogenous growth factors to the injured site improves healing outcomes significantly. Scaffold systems have the potential to provide safer, simpler bone regeneration therapies than the systems currently utilised within the clinic. ^24^ Platelets and growth factors comprise a scaffold component in the form of a fibrin matrix that could increase the bioactivity of inflammatory mediators to accelerate wound healing. Platelets and leukocyte cytokines are essential in biomaterial biology, and the fibrin matrix that supports it is the determining factor responsible for increasing the potential use of PRF. The primary source of angiogenesis is derived from fibrin gel. The fibrin matrix supports injured tissues, which affects the metabolism of epithelial cells and fibroblasts. Additionally, PRF acts as a physiological fibrin matrix, functioning as cell stems and allowing the remodeling of fibrin into being more dense connective tissue resistant. Therefore, the PRF membrane simultaneously increases the angiogenesis and the thickness or closure of the epithelium necessary for cutaneous healing. In this way, the use of platelet concentrate autologous is a promising application in the field of periodontal tissue regeneration and the alveolar bone that can be utilised in clinical circumstances that require quicker healing. ^25^

MMP-13 has been widely used as a marker and therapy in the medical field. MMP-13, an important member of the MMPs family, performs well-measured functions through degradation of type II collagen in articular cartilage and bone in osteoarthritis, concerns the molecular metastases of oral cancer, and should alter vasculogenic mimicry and endothelial-dependent vessel formation in large cell lung cancer. ^26^^–^^28^ Similar studies using the expression of MMP-13 have proven that MMP-13 plays a role in every phase of wound healing by making modifications to the wound matrix, enabling cell migration essential in the remodeling process. MMPs are responsible for collagen degradation. Tissue engineering is one of the helpful endeavours in recovering periodontal tissues and alveolar bone. The three fundamental parts in tissue engineering are progenitor cells, scaffolds and growth factors. Proteinases play an essential role in wound healing by controlling cell-grid connections and the accessibility of bioactive particles. In the research conducted by Toriseva M. *et al.* (2012), the role of matrix MMP-13 on granulation tissue development was significantly reduced in mice. Granulation tissue in MMP-13/− mice exhibited delayed myofibroblast organisation, an increased microvascular density and an almost complete absence of large vessels. In addition, the gene expression profile identified genes that were differentially expressed in the granulation tissue of MMP-13/− mice involved in biological functions, including inflammatory response, angiogenesis, cell movement, cell growth, proliferation and proteolysis. This study exhibited the role of MMP-13 in wound healing by organising cell exercises that are significant in the growth and maturation of granulation tissues, including myofibroblast work, inflammation, angiogenesis and proteolysis. ^29^

HA applications are widely used for wound healing, even the modifications continue to be developed. Hassan HH *, et al.* (2021) states that following compositional modification, cellulose acetate nanofibers containing modified HA for wound healing utilization demonstrated a high degree of response with proliferation and growing behaviors. ^30^ Samadian HA, *et al.* (2018) used HA modification dressing that resulted in the highest collagen synthesis, re-epithelialization, neovascularization, and cosmetic appearance, according to histological and histomorphometric examinations of the wounds. ^31^ Wardhana AS, *et al.* use ellagic-hydroxyapatite acid to promotes osteogenesis in bone defects by increasing the amounts of osteoblasts and therefore the expression of osteoprotegerin and osteocalcin. ^32^ HA is a scaffold that can bond with MMPs and reduce its activities in vitro. Secretion and MMP activities are regulated well, and in normal tissues, MMP is expressed on a fundamental level. If the tissue remodeling phase is necessary, such as in wound healing, MMP can be expressed and activated easily. A few diverse cell types communicated MMPs inside the skin (keratinocytes, fibroblasts, endothelial cells and inflammatory cells, such as monocytes, lymphocytes and macrophages). MMP expression can be prompted to see different signs, including cytokines, hormones and contact with other cell types or with ECM. ^3^ MMP-13 cleaves Connective Tissue Growth Factor (CTGF) and releases deliveries a few parts, which are more strong than the parent particle to actuate fibrosis. ^33^

The study's limitation is that the evaluation was done solely by looking at the level of MMP-13 immunoexpression. Further research will be required to assess the changes in the alveolar bone using intra-oral radiographic examination. Further research can also be carried out with a longer observation period to estimate the expression of other molecular markers of the periodontal tissue and alveolar bone and are also needed to confirm the effective dose of the biomaterial used when ready for use in human clinical trials.

## Conclusion

In conclusion, the administration of PRF and HA was capable of reducing the MMP-13 expression that significantly accelerate the socket healing process. This study that used platelet concentrates as growth factors and hydroxyapatite as scaffold is the best combination and can be utilised as an alternative therapy. Moreover, hydroxyapatite is an alloplastic material that has inherent bioactive properties that support osteoconduction, can bind MMPs, and showed faster healing results based on the observation time as documented by immunohistochemistry.

## Author contributions

MMD and MR conceived the plan of this research. MMD wrote the manuscript. MMD analysed the data and made the figures. MMD edited the manuscript. MMD is responsible for research in the animal laboratory, MR did the analysis of MMP-13 evaluation, and MMD supervised the whole research. All authors revised and approved the manuscript for final submission.

## Data availability

### Underlying data

Figshare: Underlying data for ‘Pre-Clinical Study: Immunohistochemical evaluation of matrix metalloproteinase-13 on Rabbit (Oryctolagus cuniculus) socket healing after application of platelet-rich fibrin with and without hydroxyapatite’

Histopathological of IHC MMP-13

https://doi.org/10.6084/m9.figshare.16529847. ^22^

This project contains the following underlying data:

= MMP-13 C 3 100×.tiff (immunoexpression of MMP-13 control group in Day 3 observation at 100× magnification).
= MMP-13 C 3 400×.tiff (immunoexpression of MMP-13 control group in Day 3 observation at 400× magnification).
= MMP-13 C 7 100×.tiff (immunoexpression of MMP-13 control group in Day 7 observation at 100× magnification).
= MMP-13 C 7 400×.tiff (immunoexpression of MMP-13 control group in Day 7 observation at 400× magnification).
= MMP-13 C 14 100×.tiff (immunoexpression of MMP-13 control group in Day 14 observation at 100x magnification).
= MMP-13 C 14 400x.tiff (immunoexpression of MMP-13 control group in Day 14 observation at 400× magnification).
= MMP-13 PRF 3 100×.tiff (immunoexpression of MMP-13 PRF group in Day 3 observation at 100× magnification).
= MMP-13 PRF 3 400×.tiff (immunoexpression of MMP-13 PRF group in Day 3 observation at 400× magnification).
= MMP-13 PRF 7 100×.tiff (immunoexpression of MMP-13 PRF group in Day 7 observation at 100× magnification).
= MMP-13 PRF 7 400×.tiff (immunoexpression of MMP-13 PRF group in Day 7 observation at 400× magnification).
= MMP-13 PRF 14 100×.tiff (immunoexpression of MMP-13 PRF group in Day 14 observation at 100× magnification).
= MMP-13 PRF 14 400×.tiff (immunoexpression of MMP-13 PRF group in Day 14 observation at 400× magnification).
= MMP-13 PRF+HA 3 100×.tiff (immunoexpression of MMP-13 PRF+HA group in Day 3 observation at 100× magnification).
= MMP-13 PRF+HA 3 400×.tiff (immunoexpression of MMP-13 PRF+HA group in Day 3 observation at 400× magnification).
= MMP-13 PRF+HA 7 100×.tiff (immunoexpression of MMP-13 PRF+HA group in Day 7 observation at 100× magnification).
= MMP-13 PRF+HA 7 400×.tiff (immunoexpression of MMP-13 PRF+HA group in Day 7 observation at 400× magnification).
= MMP-13 PRF+HA 14 100×.tiff (immunoexpression of MMP-13 PRF+HA group in Day 14 observation at 100× magnification).
= MMP-13 PRF+HA 14 400×.tiff (immunoexpression of MMP-13 PRF+HA group in Day 14 observation at 400× magnification).

Figshare: Histoscore of IHC MMP-13

https://doi.org/10.6084/m9.figshare.16531398. ^20^

This project contains the following underlying data:

= Histoscore C PRF PRF+HA (Multiplication of distribution and intensity of MMP-13 among group: control, PRF, and PRF+HA)
= Histoscore C 3, 7, 14 (Multiplication of distribution and intensity of MMP-13 in control group based on observation time)
= Histoscore PRF 3, 7, 14 (Multiplication of distribution and intensity of MMP-13 in PRF group based on observation time)
= Histoscore PRF+HA 3, 7, 14 (Multiplication of distribution and intensity of MMP-13 in PRF+HA group based on observation time)
= Histoscore 3, 7, 14 (Multiplication of distribution and intensity of MMP-13 among group based on observation time)

### Reporting guidelines

Figshare: ARRIVE checklist for ‘Pre-Clinical Study: Immunohistochemical evaluation of matrix metalloproteinase-13 on Rabbit (Oryctolagus cuniculus) socket healing after application of platelet-rich fibrin with and without hydroxyapatite’

https://doi.org/10.6084/m9.figshare.16640299. ^16^

This project contains the following underlying data:

= ARRIVE guidelines checklist full

Figshare: Figure of Table for ‘Pre-Clinical Study: Immunohistochemical evaluation of matrix metalloproteinase-13 on Rabbit (Oryctolagus cuniculus) socket healing after application of platelet-rich fibrin with and without hydroxyapatite’

https://doi.org/10.6084/m9.figshare.16641181. ^34^

This project contains the following underlying data:

= Table 1 (Table of histoscore difference of MMP-13 between groups)
= Table 2 (Table of differentiation of each treatment groups by observation times on Day 3, Day 7, and Day 14)

Data are available under the terms of the Creative Commons Zero “No rights reserved” data waiver (CC0 1.0 Public domain dedication).
