# Supplementary material for: Pre-Clinical Study: Immunohistochemical evaluation of matrix metalloproteinase-13 on rabbit ( Oryctolagus cuniculus) socket healing after application of platelet-rich fibrin with and without hydroxyapatite
Source: F1000Res. 2022 Aug 11;11:29. Originally published 2022 Jan 11. [Version 2] doi: 10.12688/f1000research.74094.2 (PMC9440371; doi:10.12688/f1000research.74094.2)
Supplement: Peer review history

# Peer review: Reviewer response for version 2

**DOI:** 10.5256/f1000research.137236.r147249

**Published:** 2022-09-02

**Authors:** Niswati Fatmah Rosyida

**Affiliations:** 1 Department of Orthodontics, Faculty of Dentistry, Universitas Gadjah Mada, Yogyakart, Indonesia

The authors have revised the points that must be corrected in the first review. Therefore the quality of the article has improved.

Is the work clearly and accurately presented and does it cite the current literature?

Yes

If applicable, is the statistical analysis and its interpretation appropriate?

I cannot comment. A qualified statistician is required.

Are all the source data underlying the results available to ensure full reproducibility?

Partly

Is the study design appropriate and is the work technically sound?

Yes

Are the conclusions drawn adequately supported by the results?

Yes

Are sufficient details of methods and analysis provided to allow replication by others?

Partly

Reviewer Expertise:

biomaterial; orthodontic tooth movement, orthodontics

I confirm that I have read this submission and believe that I have an appropriate level of expertise to confirm that it is of an acceptable scientific standard.

# Peer review: Reviewer response for version 2

**DOI:** 10.5256/f1000research.137236.r147248

**Published:** 2022-08-11

**Authors:** Joao Vitor dos Santos Canellas

**Affiliations:** 1 Department of Oral and Maxillofacial Surgery, Faculty of Dentistry, Rio de Janeiro State University, Rio de Janeiro, Brazil

The authors addressed each of the points made by the first revision, improving the quality of the paper considerably.

Is the work clearly and accurately presented and does it cite the current literature?

Yes

If applicable, is the statistical analysis and its interpretation appropriate?

I cannot comment. A qualified statistician is required.

Are all the source data underlying the results available to ensure full reproducibility?

Partly

Is the study design appropriate and is the work technically sound?

Yes

Are the conclusions drawn adequately supported by the results?

Yes

Are sufficient details of methods and analysis provided to allow replication by others?

Partly

Reviewer Expertise:

Evidence-based dentistry, Systematic reviews, meta-analysis, clinical trials, and Platelet-rich fibrin.

I confirm that I have read this submission and believe that I have an appropriate level of expertise to confirm that it is of an acceptable scientific standard.

# Peer review: Reviewer response for version 1

**DOI:** 10.5256/f1000research.77809.r142579

**Published:** 2022-07-04

**Authors:** Joao Vitor dos Santos Canellas

**Affiliations:** 1 Department of Oral and Maxillofacial Surgery, Faculty of Dentistry, Rio de Janeiro State University, Rio de Janeiro, Brazil

The authors make quite an effort trying to evaluate in a preclinical study the use of PRF with and without HA in socket healing. However, there are some points that need clarification.

 Comments: 

1. The title and the abstract of this study are appropriate, direct, and descriptive.
2. The authors reported that the sample size was determined using the Mead equation. However, they should describe how the sample size was determined exactly, including the values used to achieve the sample of 18 rabbits. Small sample sizes may make it difficult to determine if the outcome is a true finding or a type II error.
3. The authors reported that the animals were randomly divided into three groups, but they did not explain the method used to assign rabbits interventions. Bias arising from the randomization process can occur if the method of allocation is not adequately or not truly random. 
4. Important differences among commercial HA result from chemical proprieties and the manufacturing process. Authors should detail what HA was used in this research. Many studies reported that two similar alloplastic biomaterials could produce different clinical results. 
5. Authors should remove the sentence “…The limitation of this study is that, after tooth extraction, intervention was executed on the basis of the treatment group, and then simple stitches was performed. However, stitches can loosen up if the experimental animal is too active in moving its teeth and tongue, even making the stitches to come off.’’ From the method section. This information should be included in the discussion section.  
6. A detailed description must be reported in all studies related to PRF to increase the transparency of research. Please, provide the centrifugation device used, as well as the rotor angulation, radius at the clot, and tube characteristics. Each of mentioned parameters may influence regeneration success. 
7. Bias in the measurement of the outcomes can occur when the researcher involved in outcome analysis is not blind to the assigned interventions. Please inform if the qualified pathologist was blinded. If not, include this information in the study limitation paragraph. 
8. Authors use a surrogated outcome, "MMP-13 immunoexpression” to evaluate alveolar changes after tooth extraction. Discuss the limitations of this examination and other possible limitations of the present preclinical study.

Is the work clearly and accurately presented and does it cite the current literature?

Yes

If applicable, is the statistical analysis and its interpretation appropriate?

I cannot comment. A qualified statistician is required.

Are all the source data underlying the results available to ensure full reproducibility?

Partly

Is the study design appropriate and is the work technically sound?

Yes

Are the conclusions drawn adequately supported by the results?

Yes

Are sufficient details of methods and analysis provided to allow replication by others?

Partly

Reviewer Expertise:

Evidence-based dentistry, Systematic reviews, meta-analysis, clinical trials, and Platelet-rich fibrin.

I confirm that I have read this submission and believe that I have an appropriate level of expertise to confirm that it is of an acceptable scientific standard, however I have significant reservations, as outlined above.

# Response

**Published:** 2022-08-01

**Authors:** META DAMAYANTI

**Affiliations:** Padjadjaran University, Indonesia, Indonesia

The authors thank reviewer for the excellent review of the paper and for the in-depth suggestions made to improve the quality of the article. The followings are the responses to the recommendations of the reviewer. The valuable suggestions and corrections are incorporated cautiously, and the paper is made more transparent while revising the manuscript. All changes have been made in response to the reviewer. I have just submitted version 2. Furthermore, to answer point-by-point:

***The authors reported that the sample size was determined using the Mead equation. However, they should describe how the sample size was determined exactly, including the values used to achieve the sample of 18 rabbits. Small sample sizes may make it difficult to determine if the outcome is a true finding or a type II error.***

 The exact determined sample has been updated as suggested to avoid an error. This approach further allowed us to reduce the required number of animals according to the principle of the three R’s (replacement, reduction, and refinement) and ARRIVE 2.0 guidelines for animal handling.

***The authors reported that the animals were randomly divided into three groups, but they did not explain the method used to assign rabbits interventions. Bias arising from the randomization process can occur if the method of allocation is not adequately or not truly random.***

 We applied simple random sampling to divide into three groups of treatment and then three groups of observation time. Randomization processes for the animal study have been described in greater detail in the new version manuscript.

***Important differences among commercial HA result from chemical proprieties and the manufacturing process. Authors should detail what HA was used in this research. Many studies reported that two similar alloplastic biomaterials could produce different clinical results.***

 We agree that there are differences between commercial HA. Because we already have experience with HA. We use HA material with the brand that is most often used by dentists today, namely a modification made from the Gama-Cha brand. We recognize the limitations of the ingredients, and we agree that further research is needed to see how commercial HA administration differs from homemade biocompatible products.

***Authors should remove the sentence “…The limitation of this study is that, after tooth extraction, the intervention was executed on the basis of the treatment group, and then simple stitches was performed. However, stitches can loosen up if the experimental animal is too active in moving its teeth and tongue, even making the stitches to come off.’’ From the method section. This information should be included in the discussion section.***

 We have deleted the sentences regarding the statement above. We agree with the comment that it should be removed. This aspect has consequently been removed from the text, and more explained in the discussion section.

***A detailed description must be reported in all studies related to PRF to increase the transparency of research. Please, provide the centrifugation device used, as well as the rotor angulation, radius at the clot, and tube characteristics. Each of mentioned parameters may influence regeneration success.***

 Thank you for your helpful report. Your suggestions have allowed us to improve our manuscript and present our data more accurately. We will add to the new version paper, and more detailed documentation will also be provided.

***Bias in the measurement of the outcomes can occur when the researcher involved in outcome analysis is not blind to the assigned interventions. Please inform if the qualified pathologist was blinded. If not, include this information in the study limitation paragraph.***

 Thank you for your comment. Blind observers (pathologists) assigned random areas of IHC photographed using a Leica microscope without knowing the group identity on histopathology slides. We added this more detailed explanation to the materials and methods section. Revisions have been included in the revised text.

***Authors use a surrogated outcome, "MMP-13 immunoexpression” to evaluate alveolar changes after tooth extraction. Discuss the limitations of this examination and other possible limitations of the present preclinical study.***

 The limitations of this examination and other possible limitations of the present preclinical study have been included in the revised text.

# Peer review: Reviewer response for version 1

**DOI:** 10.5256/f1000research.77809.r119475

**Published:** 2022-02-07

**Authors:** Niswati Fatmah Rosyida

**Affiliations:** 1 Department of Orthodontics, Faculty of Dentistry, Universitas Gadjah Mada, Yogyakart, Indonesia

1. Abstract:

 - Kindly rewrite the conclusion in the abstract, because the conclusion is different from the result of this study.

 2. Method:

 - Kindly mention the production company of the material used.

 -"The animals were not selected on the basis of sex and age because they did not affect the treatment". Kindly comment on this statement. Please add the literature which proves that statement.

 - Please mention the reason not to control the age of animals?

 - Kindly describe the preparation of PRF+HA. Mention the formulation of PRF and PRF+HA which were used in this study, the concentration of each material, the dosage, and dosage form.

 - "...the immunoexpression scores of MMP-13 were evaluated by a qualified pathologist." - please describe how intra-examiner calibrations were measured.

 -"...if normally distributed, and parametric tests were performed. The evaluation of MMP-13 among the groups and observation times was performed statistically with the Kruskal–Wallis test". Please add the reason for using a nonparametric test (Kruskal Wallis).

 3. Results:

 - Kindly revise the legend of the table.

 - Please add an asterisk or similar sign to highlight where there are significant differences between the groups.

 - Kindly revise the legend of the table. Add the information of magnification.

 4. Discussion:

 - "...there was a significant difference based on the observation time from Day 3 to Day 14." - kindly add literature in paragraph two of the discussion which strengthens this result. 

Is the work clearly and accurately presented and does it cite the current literature?

Yes

If applicable, is the statistical analysis and its interpretation appropriate?

I cannot comment. A qualified statistician is required.

Are all the source data underlying the results available to ensure full reproducibility?

Partly

Is the study design appropriate and is the work technically sound?

Yes

Are the conclusions drawn adequately supported by the results?

Yes

Are sufficient details of methods and analysis provided to allow replication by others?

Partly

Reviewer Expertise:

biomaterial; orthodontic tooth movement

I confirm that I have read this submission and believe that I have an appropriate level of expertise to confirm that it is of an acceptable scientific standard, however I have significant reservations, as outlined above.

# Response

**Published:** 2022-08-01

**Authors:** META DAMAYANTI

**Affiliations:** Padjadjaran University, Indonesia, Indonesia

We want to thank the invited reviewer for their valuable comments and suggestions. We feel that the scientific value of our manuscript has increased significantly. This revised version of the manuscript includes the following significant adjustment as suggested by the reviewer. We have proofread the manuscript a couple of times. Thank you for your review. I have just submitted version 2. To answer point-by-point:

***Abstract:***

***- Kindly rewrite the conclusion in the abstract, because the conclusion is different from the result of this study.***

 A revised abstract about the conclusion has been added in the new versions.

***Method:***

***- Kindly mention the production company of the material used.***

 References for the product of material used have been added in the revised version. Also, we generated and utilized the commonly used product.

***-"The animals were not selected on the basis of sex and age because they did not affect the treatment". Kindly comment on this statement. Please add the literature which proves that statement.***

 Thank you for making us aware of this. We followed your suggestion, and we added this to the script. As in all studies here, we mentioned that gender had no effect because we assumed no hormonal influence on oral wound healing. However, I will add gender-specific criteria because our study used male and adult animal models. However, for age, we have revised it by adding an age range as an inclusion criterion.

***- Please mention the reason not to control the age of animals?***

 Thanks for mentioning this. I missed a clear statement of inclusion criteria, and a more detailed age range will also be corrected.

***- Kindly describe the preparation of PRF+HA. Mention the formulation of PRF and PRF+HA which were used in this study, the concentration of each material, the dosage, and dosage form.***

 Thank you for letting us know that this explanation has gotten lost somehow. The methodology has been clarified, such as formulations of PRF and PRF+HA, the concentration of each material, the dosage, and the dosage form used in the study. Materials and methods have been described more accurately as suggested in the new versions.

***- "...the immunoexpression scores of MMP-13 were evaluated by a qualified pathologist." - please describe how intra-examiner calibrations were measured.***

 Evaluation by a qualified pathologist has been described as well as how intra-examiner calibration is measured, including several points to address the importance of this research study evaluation method.

***-"...if normally distributed, and parametric tests were performed. The evaluation of MMP-13 among the groups and observation times was performed statistically with the Kruskal–Wallis test". Please add the reason for using a nonparametric test (Kruskal Wallis).***

 In the revised text, we have corrected the error of the normal to abnormal distribution, so that a non-parametric test with the Kruskal-Wallis test was performed. Because in this study we want to examine differences in stratified groups. Additions suggested by reviewers have also been added detailed in the methods section.

***Results:***

***- Kindly revise the legend of the table.***

 Thank you for your detailed comment. We replaced the revised table in the new version paper.

***- Please add an asterisk or similar sign to highlight where there are significant differences between the groups.***

 Thank you for the suggestions. We have been adding a similar sign to highlight significant differences between the groups and are now uniform throughout the manuscript to increase readability.

***- Kindly revise the legend of the table. Add the information of magnification.***

 The legend of the table and information on magnification has been fully addressed in the revised text.

***Discussion:***

***- "...there was a significant difference based on the observation time from Day 3 to Day 14." - kindly add literature in paragraph two of the discussion which strengthens this result.***

 The comment in the discussion section to strengthen the result has been addressed in the revised text by providing more literature.
